# A Removal of Eye Movement and Blink Artifacts from EEG Data Using Morphological Component Analysis

**DOI:** 10.1155/2017/1861645

**Published:** 2017-01-17

**Authors:** Balbir Singh, Hiroaki Wagatsuma

**Affiliations:** ^1^Graduate School of Life Science and Systems Engineering, Kyushu Institute of Technology (KYUTECH), Kitakyushu, Japan; ^2^RIKEN Brain Science Institute, Wako, Japan; ^3^Artificial Intelligence Research Center, National Institute of Advanced Industrial Science and Technology (AIST), Tsukuba, Japan

## Abstract

EEG signals contain a large amount of ocular artifacts with different time-frequency properties mixing together in EEGs of interest. The artifact removal has been substantially dealt with by existing decomposition methods known as PCA and ICA based on the orthogonality of signal vectors or statistical independence of signal components. We focused on the signal morphology and proposed a systematic decomposition method to identify the type of signal components on the basis of sparsity in the time-frequency domain based on Morphological Component Analysis (MCA), which provides a way of reconstruction that guarantees accuracy in reconstruction by using multiple bases in accordance with the concept of “dictionary.” MCA was applied to decompose the real EEG signal and clarified the best combination of dictionaries for this purpose. In our proposed semirealistic biological signal analysis with iEEGs recorded from the brain intracranially, those signals were successfully decomposed into original types by a linear expansion of waveforms, such as redundant transforms: UDWT, DCT, LDCT, DST, and DIRAC. Our result demonstrated that the most suitable combination for EEG data analysis was UDWT, DST, and DIRAC to represent the baseline envelope, multifrequency wave-forms, and spiking activities individually as representative types of EEG morphologies.

## 1. Introduction

The electrophysiological mechanism of how EEG signals are generated and information of what they represent still remain unclear and the most plausible hypothesis is that the signals are composed of synchronous spiking activities with respect to the oscillatory modulation of the local field potential [1]. Therefore, EEGs have been used as an index to represent the brain state, such as being awake, sleeping, and selective attention, and to estimate which brain regions are active in comparison with other regions if they are located on the superior surface of the brain close to the cranial bone, like part of the cerebrum. The most difficult issue in the data analysis of EEGs is the uncertainty of the discrimination of the signal and noise. Biological signals contain multiple types of the signals caused by different internal mechanisms, such as EOG (electrooculogram) generated by the movement of eyeballs and eyelids and EMG (electromyogram) generated by muscular movements of body parts. Unless individual electrophysiological mechanisms can be isolated, the problem of the impossibility in pursuit of the true signal is inevitable. The EEG has been known as the most noninvasive tool in particular for clinical diagnosis and neuroscience research, while medical professionals and researchers in the related fields have the difficulty of the signal contamination. In the engineering field, EEGs are used practically in brain-computer-interface technology [2–10]. In those cases, the most serious artifact is ocular related potential, for example, eye movements and eye blinks, and thus methods of artifact removal have been proposed [11–13]. The proposed method mostly dealt with linear and stationary signal decomposition for artifact removals. However, there are a few methods to treat nonlinear and nonstationary properties in EEGs [14–16]. EEG signals decomposition indicates that traditional methods are not simply applicable to nonlinear and nonstationary signals in the purpose of artifact removals [17].

Recently, signal decomposition by focusing morphological components is attracting more and more attention due to its applicability to nonlinear and nonstationary signal properties [18–20]. Originally, signal feature extractions using linear analysis in time-frequency domains had been studied via Fourier and wavelet transforms or eigenvectors and subspace theories in the simplest manner [21]. The blind source separation [22] has been discussed widely on the issue of a linear mixture signal, and Independent Component Analysis (ICA) and Principal Component Analysis (PCA) are representative methods. In the case of the EEG signal decomposition, those methods were frequently applied [23, 24], especially in the offline analysis. In PCA, the EEG components are decomposed on space/time basis, while, as disadvantage, it is difficult to reconstruct overall signals by the linear combination of principal components (PCs) because of the ignorance of signals with small amplitudes and irregular changes. Therefore, the accurate reconstruction in those methods requires the prior and detailed knowledge to identify PCs corresponding to artifacts [25, 26]. The limitation led a shift of the research trends from PCA to ICA with high order statics to specify independence in the signal. On the other hand, since the ICA is restricted to the measure of statistical independence, ICA faces the difficulty of detecting signal components if Gaussian noise is contaminated in the manner that the noise spreads over in an undesired way into the signal components [11–13, 27, 28].

In plausible EEG decomposition [29], the key role is the effectiveness in analyzation, enhancement, and synthetization of signal properties, including the nonlinear and nonstationary changes. Blind source separation, such as ICA, has demonstrated the decomposition performance even in complex signals; however the sparsity is getting to be highlighted as an extended concept because of the consistency between signal analysis and synthesis in a systematic manner [30] and then the methodology based on the sparsity by using redundant transforms was introduced for signal decomposition in various applications [31]. MCA is one of the methods. In terms of MCA, the sparsity plays a vital role in separating different time/frequency properties or morphologies of individual signal components, which were demonstrated in the recent studies [18, 32, 33]. The effectiveness of the MCA based noise removal was mostly clarified in image processing [19, 20, 31, 34]. However, we hypothesized that the MCA decomposition is effective in the EEG artifact removal and it clarifies which kinds of signal morphologies contaminate the signal as true biological signals, by using redundant transform or mixed overcomplete dictionary in the sense of MCA [35]. Different dictionaries which mean different types of mathematical basis function represent evoked potentials generated by different electrophysiological mechanisms. Yong et al. [36] preliminarily reported the effectiveness in the EEG artifact removal and provided a less comprehensive analysis with MCA in the framework of verification of how EEG true signal preserved after noise removals even with various EOG fluctuations [37].

In the present study, we proposed an EEG decomposition method based on the sparsity and overcompleteness dictionary by specifying the best combination of dictionaries [31] and discussed the reason in the sense of the EEG frequency properties. Depending on the set of dictionaries, reconstructed signals were highly different in the representation of time/frequency features in signal [35, 38, 39]. In the computer experiment, we used the Block-Coordinate-Relaxation (BCR) algorithm to minimize error in signal reconstruction and obtain the sparsest representation of desired features. The goal of this study is to propose the systematic way of the artifact removal in EEG signals by employing MCA and specify time/frequency properties to represent signal components by verifying the appropriate combination of the dictionaries.

## 2. Methods and Materials

### 2.1. Decomposition Method

Numerous methods have been commonly formulated as the linear combination to suppress or remove the artifacts from EEGs. If a signal and a noise are linearly independent, the noise can be removed by replacing coefficients representing the noise part with zero when the whole signal is reconstructed. The blind source separation methods, like ICA and PCA, commonly used the BCI system to decompose the EEG signal [11–13, 23, 27–29, 40–42] as follows:

(1) S=Φ×X,Y=W×S.

The recorded EEGs from electrodes attached to the scalp (abbreviated as scalp EEG) *S* can be given by (1), where *X*(*t*) = [*x*_1_(*t*), *x*_2_(*t*),…, *x*_*k*_(*t*)] is time series of coefficients called signal components or components simply and Φ is the mixing matrix to determine the way to split *S* between the signal and noise. In ICA decomposition, the final target is to find the mutual independence *W* matrix that satisfies *W* = Φ^−1^ and each row vector in *Y*, unmixing matrix, is approximately equal to a scaled value of one row vector in *X*. The signal then is decomposed into EEGs assumed as the set of true signals and artifacts components. The decomposition methods conventionally require prior knowledge about properties of the target components coupling with the constraints [29], as discussed in the Introduction. A heuristic factor remains to be an obstacle for the full automation of the signal decomposition.

### 2.2. EEG-EOG Component Morphology

The cerebral cortex is located in the outer region of brain hemispheres just beneath the skull bone and, therefore, the activities are accessible by electrical potentials recorded evenly on the scalp. These cortical regions are locally separated depending on functions, such as decision-making function (frontal cortex), motor control (premotor cortex), body sensations (somatosensory cortex), and the processing of the sensory inputs in vision and audition (primary visual and auditory cortex), and then potentials from different positions on the scalp contain information of neuronal activities in different cortices if signals are clearly separated from each other and from artifacts. The production of other electric potentials from muscular, eyeball, and eyelid movements contaminates the scalp EEG in an evitable manner of leaking potentials in the electrophysiological system connecting the brain and muscular-skeletal system. On the other hand, different biological systems have different electrophysiological properties and the nature will be the key to solve the complex decomposition problem. As the traditional knowledge in the medical field [43], it is known that EEG signals have specific characteristics on the shape of the waveform called morphology: “monomorphic,” “polymorphic,” “sinusoidal,” and “transient” types are recognized differently based on characteristics of a single dominant activity, multiple frequencies forming complex activity, sine wave-like activity, and spikes and/or sharp waves (spikes in a duration of 20–70 msec and sharp waves with a pointed peak and 70–200 msec duration). If it is possible to decompose the recorded EEG with respect to those morphologies of interest, this brings us a large benefit because it leads the way to the “true” EEGs.

**Figure 1:**
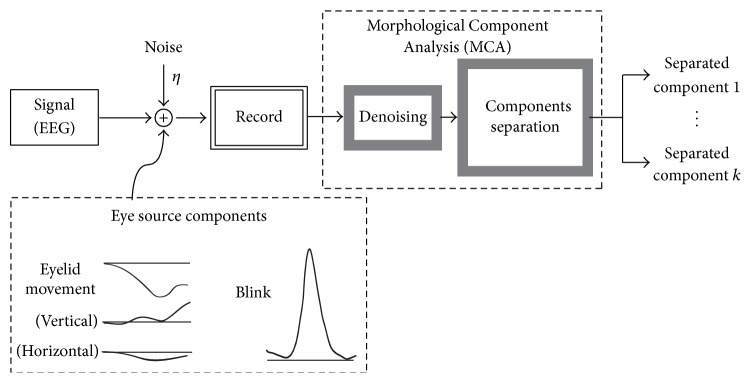
A schematic diagram of the proposed method for morphological component analysis for the EEG-EOG signal separation.

According to the electrophysiological mechanism in the nervous system coupled with myogenic potential evoked by ocular movements [44, 45], the rotation of an eyeball generates potential with an amplitude depending on the degree of the rotation [46], which is known as the corneoretinal dipole and observed as the staying potential of approximately 500 *μ*V as maximum from the EOG recording in the 4–20 Hz range [47]. The phenomena had been investigated via the studies of saccade movements [28, 48–50]. As mentioned above, EEG and EOGs potential have specific morphologies. Morphologies of eye movements and eye blinks can be considered as slow change with respect to the EEG time scale and have a bump shape with a large peak amplitude [51, 52]. Since the presence of repetitive peaks frequently appears in the diagnosis of epilepsy [53], we assumed the single bump is the typical eye blink and assumed the multiple types of slow baseline changes are eyeball rotations, as schematically shown in Figure 1.

### 2.3. Decomposition Using Morphological Component Analysis

Recently, decomposition of components in image and time series has a large expectation in applications, such as minimizing of the data size for transferring the data via the Internet. MCA based methods fit for the purpose and have the advantage in the accurate reconstruction of the original data after noise removal, which relies on the sparsity and overcompleteness of the dictionary. In the theory of MCA, the overcomplete dictionary is represented by Φ ∈ *R*^*n*×*k*^, where *k* is the morphological component of signal for {*ϕ*_*k*_}_*k*∈Γ_, where Γ is the index set of dictionaries. A mixed EEG signal *S* ∈ *R*^*n*^ can be represented as a sparse linear combination of the coefficient. According to Chen et al. (2001) [38], the overcomplete dictionary Φ is a set of redundant transforms, which are defined by a set of mathematical functions to represent the specific morphologies. In the process to obtain the final set of coefficients for accurate reconstruction of the original signal, the sparseness of the coefficient matrix is crucial. In the theory, there exists a dictionary that can reproduce the specific features of the signal if the appropriate iteration method is introduced to pursue the unique sparse representation. The concept of sparsity and the overcompleteness dictionary has theoretically extended the traditional signal decomposition to feature extractions focusing on multiple types of morphologies simultaneously. Due to selection freedom of dictionaries, the signal can be decomposed with explicit dictionary [38] and sometimes it cannot be decomposed in the other form of dictionaries. A dictionary is defined as collection of waveforms {*ϕ*_*k*_}_*k*∈Γ_, and the input signal *S* is assumed to be reconstructed by a linear combination of a set of bases' elements *ϕ*_*k*_, and then the signal *S* is expressed as a single vector of *S* ∈ *R*^*N*^ and satisfies *S* = *s*_1_ + *s*_2_,…, *s*_*K*_, where *s*_1_, *s*_2_,…, *s*_*k*_ are subcomponents, that is, different morphologies. We employed this system to record EEG signal *S* as shown in Figure 1. The approximate decomposition of signal *S*′ into its building components can be expressed as

(2) S∑i=1kβiϕi+ζ=β1ϕ1+β2ϕ2+⋯+βkϕk+ζ≅s1+s2+⋯+skζ≪1S′.

Therefore *β* is the target coefficients for reconstruction of the original EEG signal based on the assumption *ζ* ≪ 1, which means that the remainder *ζ* is negligibly small. In the consideration that *ζ* represents the noise part, (2) without noise can be written as

(3) $$\begin{array}{c} S^{\prime }=\overset{k}{\underset{i=1}{\sum }}\beta_{i}\phi_{i}=\beta \Phi . \end{array}$$

Equation (3) is consistent with (1). The problem to solve is how optimized coefficients can be derived, and the equation is rewritten as follows:

(4) β1opt,β2opt,…,βkopt=arg minβ1,…,βk ∑i=1kβi0subject  to: S′=∑i=1kβiϕi.

The problem is how the MCA concept can be embedded in the systems to decompose biomedical signal especially for EEG signal. In this formulation, time-frequency parameters are totally consistent with traditional decomposition methods which can be applied to the biomedical signal decomposition, such as PCA, wavelets, and ICA, in the sense of the single set of bases. One advantage of MCA is the availability of the combination of multiple basis functions, including traditional basis like wavelet decomposition as a part of the component, called redundant transforms. Thus, MCA is expected to reveal what kind of the specificity exists in time-frequency properties of EEG data. Concrete problems in this viewpoint can be addressed as (a) what is the best combination of dictionaries of MCA for the EEG decomposition? and (b) what is the true EEG signal in the form of obtained sparsest representation based on selected dictionaries *ϕ*_*k*_?.

**Figure 2:**
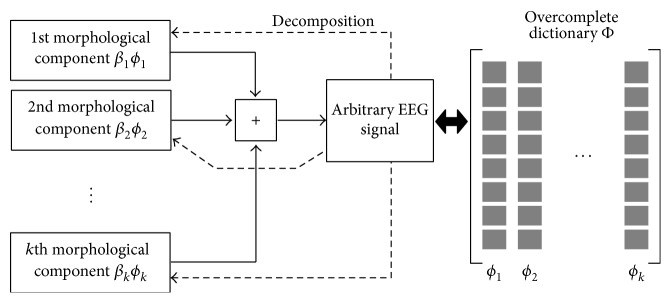
A schematic diagram for EEG signal decomposition using explicit dictionary.

Figure 2 schematically illustrates the MCA decomposition process of arbitrary EEG signal that is assumed to be a linear combination of *k* morphological component to be decomposed using explicit dictionaries.

In the assumption of three types of dictionaries (*k* = 3), the following three cases are considerable by focusing on individual dictionaries.

**Case 1 .**

An overcomplete dictionary *ϕ*_1_ is representing the component *s*_1_, *ϕ*_1_ ∈ *M*^*N*×*L*_1_^, where *N* ≫ *L*_1_, *N* being the number of samples, that is, the number of time points in the recorded data.

(i) For *s*_1_, *β*_1_^opt^ = arg min_*β*_‖*β*‖_0_ subject to *s*_1_ = *ϕ*_1_*β*, while solving this equation leads to the sparse solution (‖*β*_1_^opt^‖_0_ < ‖*β*_12_^opt^‖_0_, ‖*β*_13_^opt^‖_0_).
(ii) For *s*_2_, *β*_12_^opt^ = arg min_*β*_‖*β*‖_0_ subject to *s*_2_ = *ϕ*_1_*β*, while solving this equation leads to nonsparse solution.
(iii) For *s*_3_, *β*_13_^opt^ = arg min_*β*_‖*β*‖_0_ subject to *s*_3_ = *ϕ*_1_*β*, while solving this equation also leads to nonsparse solution.

**Case 2 .**

An overcomplete dictionary *ϕ*_2_ is representing the component *s*_2_, *ϕ*_2_ ∈ *M*^*N*×*L*_2_^, where *N* ≫ *L*_2_.

(i) For *s*_2_, *β*_2_^opt^ = arg min_*β*_‖*β*‖_0_ subject to *s*_2_ = *ϕ*_2_*β*, while solving this equation leads to the sparse solution (‖*β*_2_^opt^‖_0_ < ‖*β*_23_^opt^‖_0_, ‖*β*_21_^opt^‖_0_).
(ii) For *s*_3_, *β*_23_^opt^ = arg min_*β*_‖*β*‖_0_ subject to *s*_3_ = *ϕ*_2_*β*, while this equation also has nonsparse solution.
(iii) For *s*_1_, *β*_21_^opt^ = arg min_*β*_‖*β*‖_0_ subject to *s*_1_ = *ϕ*_2_*β*, while this equation also has nonsparse solution.

**Case 3 .**

An overcomplete dictionary *ϕ*_3_ is representing the component *s*_3_, *ϕ*_3_ ∈ *M*^*N*×*L*_3_^, where *N* ≫ *L*_3_.

(i) For *s*_3_, *β*_3_^opt^ = arg min_*β*_‖*β*‖_0_ subject to *s*_3_ = *ϕ*_3_*β*, while solving this equation leads to the sparse solution (‖*β*_3_^opt^‖_0_ < ‖*β*_32_^opt^‖_0_, ‖*β*_31_^opt^‖_0_).
(ii) For *s*_2_, *β*_32_^opt^ = arg min_*β*_‖*β*‖_0_ subject to *s*_2_ = *ϕ*_3_*β*, while solving this equation leads to nonsparse solution.
(iii) For *s*_1_, *β*_31_^opt^ = arg min_*β*_‖*β*‖_0_ subject to *s*_1_ = *ϕ*_3_*β*, while solving this equation leads to nonsparse solution.

Theoretically speaking, by using three dictionaries MCA can divide the signal into components depending on each dictionary of *ϕ*_1_, *ϕ*_2_, and *ϕ*_3_ as the sparest representation of all signals. The sparse components are described mathematically as

(5) β1opt,β2opt,β3opt=arg minβ1,β2,β3 β10+β20+β30subject  to: S=β1ϕ1+β2ϕ2+β3ϕ3.

This formulation states a nonconvex optimization problem to separate the components of the signal. However, each *ϕ*_*k*_ needs to be efficient in a specific component, while it remains noneffective in other signal components. The nonconvex optimization problem indicated that it is difficult to solve (5) in a simple manner and then the Basis Pursuit (BP) method [54] was proposed based on the idea that the replacement of *l*^0^ norm by *l*^1^ norm results in the error minimization. According to the improvement, the BP [54] was successfully formulated into an accurate method to represent the sparest of components, which is described as

(6) $$\begin{array}{c} \left\{\;\beta_{1}^{opt},\beta_{2}^{opt},\beta_{3}^{opt}\;\right\}=\underset{\beta_{1},\beta_{2},\beta_{3}}{arg\;min}\overset{3}{\underset{i=1}{\sum }}{\left\|\;\beta_{i}\;\right\|}_{1}+\lambda {\left\|\;S-\sum_{i=1}^{3}\phi_{i}\beta_{i}\;\right\|}_{2}^{2}. \end{array}$$

In this system, *l*^2^ norm is considered to be the error norm based on the assumption that the residual acts as a white zero-mean Gaussian noise and another important finding is the representation of noise models *l*^1^ Laplacian noise with the consideration of *l*^*∞*^ uniformly distributed noise, in the form of the optimization problem. *λ* represents the stopping criterion or threshold. By using the Block-Coordinate-Relaxation (BCR) method [55], the optimization problem can be solved in finite computation time. The procedure is given below:

(1) Initialize = *I*_max_; number of iterations = *L*; threshold: *δ* = *λ∗I*_max_.
(2) Perform *L* times:
Part (1): update *s*_1_, assuming *s*_2_ and *s*_3_ are fixed.
  (a) Calculate the residual *R* = *S* − *s*_2_ − *s*_3_
  (b) Calculate *β*_1_ = *ϕ*_1_^*T*^*R*
  (c) Threshold the coefficient of *β*_1_ and obtain $\hat{\beta_{1}}$
  (d) Reconstruct *s*_1_ by $s_{1}=\phi_{1}\hat{\beta_{1}}$
Part (2): update *s*_2_, assuming *s*_1_ and *s*_3_ are fixed.
  (a) Calculate the residual *R* = *S* − *s*_1_ − *s*_3_
  (b) Calculate *β*_2_ = *ϕ*_2_^*T*^*R*
  (c) Threshold the coefficient of *β*_2_ and obtain $\hat{\beta_{2}}$
  (d) Reconstruct *s*_2_ by $s_{2}=\phi_{2}\hat{\beta_{2}}$
Part (3): update *s*_3_, assuming *s*_1_ and *s*_2_ are fixed.
  (a) Calculate the residual *R* = *S* − *s*_1_ − *s*_2_
  (b) Calculate *β*_3_ = *ϕ*_3_^*T*^*R*
  (c) Threshold the coefficient of *β*_3_ and obtain $\hat{\beta_{3}}$
  (d) Reconstruct *s*_3_ by $s_{3}=\phi_{3}\hat{\beta_{3}}$
(3) Update the threshold by *δ* = *δ* − *λ*.
(4) If *δ* > *λ*, return to Step (2), else finish.

## 3. Hypothesis

In the present study, we hypothesized that appropriate three dictionaries of MCA specifically for EEG recording data were undecimated wavelet transform (UDWT), discrete sine transform (DST), and DIRAC (aka standard unit vector basis, or Kronecker basis) Fadili et al. [17]. UDWT contributes to separating slow and bump morphologies for EOG and EEG transient slow changes, DST is for monomorphic and polymorphic EEG components (major EEG parts), and DIRAC is for spike type activities in transient EEGs. In comparison in the simulated experiment, we used the discrete cosine transform (DCT), discrete sine transform (DST) [56, 57], and local discrete cosine transform (LDCT) dictionaries for major EEG parts. For the verification of the hypothesis, the intracranial EEG (iEEG) data was assumed to be “true EEG” signal, which was recorded from the real brain activity, and artificial EOGs including bump and slow changes were introduced and the performance of the accurate reconstruction of the true EEGs was examined. In iEEG, there are two types of the data under the conditions of eye-closing and eye-opening 4.2. According to the neuroscientific evidence, EEGs have a clear peak in the low-frequency range around 10 Hz in the frequency spectrum under the eye-closing condition 4.2. In the Results, correlation coefficient (cc) was used for the validation of accuracy in reconstruction and the frequency spectrum was for the validation of the nature of the information contained in EEGs.

## 4. Results

For verification of our hypothesis in the computer experiments, three types of the data were used, (1) all simulated data, (2) a combination of real iEEG and simulated EOG, and (3) recording of real EEG-EOG data, and our proposed method was validated.

### 4.1. Simulated Data

**Figure 3:**
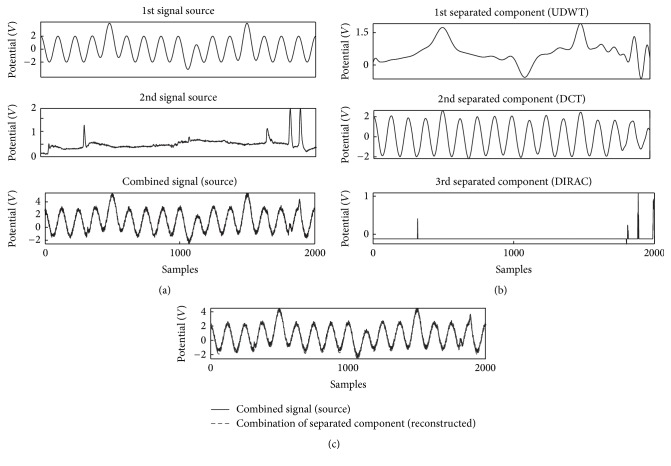
An example of simulated signal for decomposition and (a) the cosine with bump and spikes signals; and combined signal with white noise (*η* = 20%), (b) separated components with explicit dictionaries UDWT-DCT-DIRAC, and (c) comparison between combined signal and sum of separated components (cc = 0.99).

In the first place, two simulated signal sources were prepared for the simple test of the proposed method. Initially, Yong et al. [36] proposed a combination of the wavelet, DCT, and DIRAC for EEG artifact removals, while their results could not tell how effective the method was in a qualitative manner. In our experiment, the first source signal consisted of a cosine wave, which was assumed to be a monomorphic EEG signal, and the second source consisted of blinks component with three bumps designed as usual EOG signals. The simulated signal as a mixture of the two sources and white noise (*η* = 20%) was shown in Figure 3(a), where *η* was defined as the percentage of the maximum amplitude of the input signal. Our proposed MCA method was applied to separate the components from the simulated signal with the explicit dictionaries UDWT, DCT, and DIRAC as shown in Figure 3(b), as a replication test. The correlation coefficient between the simulated signal and the sum of all components was higher than 0.99 and the simulated result proved the accuracy of decomposed components by MCA with UDWT, DCT, and DIRAC explicit dictionaries (Figure 3(c)).

### 4.2. Simulated EOG Contaminated iEEG Signal

The previous section was a simple example of the simulated data. In this section, we introduced a new validation way to test the proposed method in a qualitative manner. The real iEEG signals were obtained under the closing eye condition. The linear combination of simulated EOG and real iEEG signals was used for the test. In this case, we assumed that iEEG had already included a usual level of the white noise and then did not add further noise additionally. The iEEG dataset was given by Andrzejak et al. [58] with 100 trials, and the sampling rate was at 173.61 Hz (0.00576 s/sample) and 2^10^(=1024) samples took about 6 s (5.89824 s). The linear combination of simulated EOG and iEEG signals was applied for the validation.

**Figure 4:**
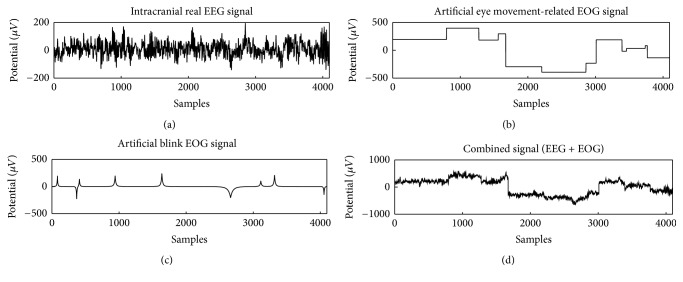
A systemic representation of different morphological signals: (a) intracranial EEG signal, (b) artificial block EOG signal, (c) artificial blink EOG signal, and (d) combined signal.

There were different combinations for simulated EOG as assumed: artificial eye movement, which was considered as the step function, and eye blinks by bump signal. The flatness signals with elevations with slow time scales compared with the EEG time scale represent the gaze-type eyeball rotations. The signals can be reconstructed by a mathematical method defined by the rate of change ${\left(\hat{g}\right)}^{\prime }$ of $\hat{g}$ which satisfies that $\left\{t|{\left(\hat{g}\right)}^{\prime }>0\right\}$ should be 0. Thus, the definition of the EOG smoothness is described as

(7) $$\begin{array}{c} {\left(\;\hat{g}\;\right)}_{I}^{\prime }=\left\{\begin{array}{cc} {\left(\hat{g}\right)}^{\prime }\left(t\right) & {\left(\hat{g}\right)}^{\prime }\left(t\right)=0\\ 0 & {\left(\hat{g}\right)}^{\prime }\left(t\right)>0, \end{array}\right. \end{array}$$

where $I=\{t|{\left(\hat{g}\right)}^{\prime }=0\}$ leads to $(\hat{g}{)}_{I}^{\prime }\equiv 0$ according to its definition as shown in Figure 4(b). In addition, the bumps signal was considered as the blink type EOG signal shown in Figure 4(c).  Figure 4(d) showed the schematic example for the semisimulated signal. In the same way, 100 datasets of semisimulated signals with a random combination of components in time series were used for the validation.

**Figure 5:**
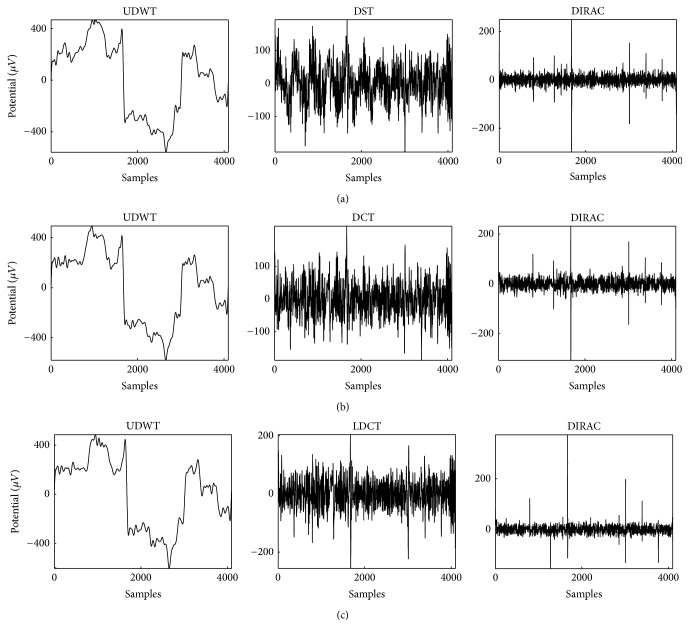
Components separation by MCA: (a) explicit dictionaries are UDWT, DST, and DIRAC. (b) UDWT, DCT, and DIRAC. (c) UDWT, LDCT, and DIRAC, respectively, at *λ* = 4. The original signal for decomposition was shown in Figure 4(d) as combined signal.

Figures 5(a), 5(b), and 5(c) demonstrated a set of results for the decomposition of the semisimulated signal by explicit dictionaries, depending on the combination of dictionaries. As mentioned in Section 2.3, the stopping criterion depended on *λ∗*threshold and the parameters in this comparative study used a different combination of explicit dictionaries (UDWT-DCT-DIRAC, UDWT-DST-DIRAC, and UDWT-LDCT-DIRAC) and different type of thresholds, either hard and soft, and *λ* value varied from 3 to 5.

**Figure 6:**
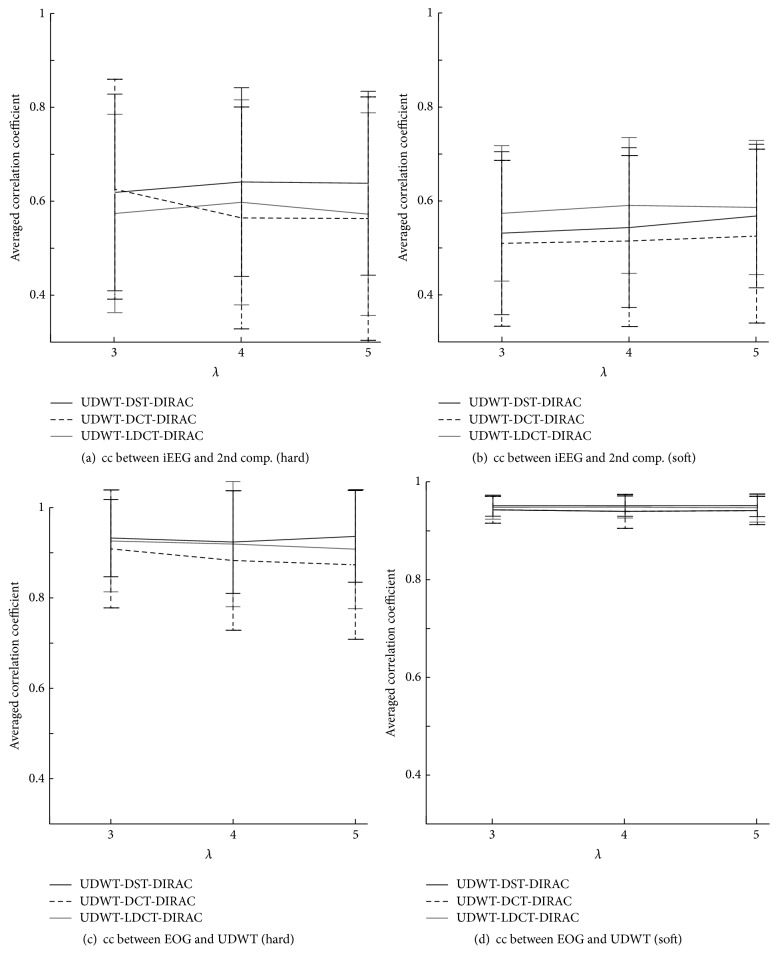
A comparison between cc of decomposed morphological component with iEEG signal and artificial EOG with hard and soft thresholds. The mean value and standard deviation were calculated from all 100 decomposed datasets by explicit dictionaries. (a) Second morphological component was decomposed by DST, DCT, and LDCT with hard and (b) soft threshold, respectively. (c) First morphological component was decomposed by UDWT with a hard threshold and (d) soft threshold, respectively. 100 trials of iEEG and artificial EOG were used.

Figures 6(a) and 6(b) showed the averaged cc of decomposed component by a respective combination of explicit dictionaries with hard and soft thresholds. For EEG signal decomposition performance, cc between iEEG and either DST, DCT, or LDCT component was evaluated depending on the three combination types (Figure 6(a)). For EOG signal decomposition performance, cc between EOG and UDWT component was evaluated (Figure 6(b)). In comparison between hard and soft thresholds, the average value of the hard threshold is around 0.6 which is larger than that in the soft threshold, while the average value of the soft threshold is around 0.95 demonstrating fewer variances than those in the hard threshold. This method indicates that UDWT dictionary with a soft threshold is the stable performance according to the fitness of the morphological property in this case.

**Figure 7:**
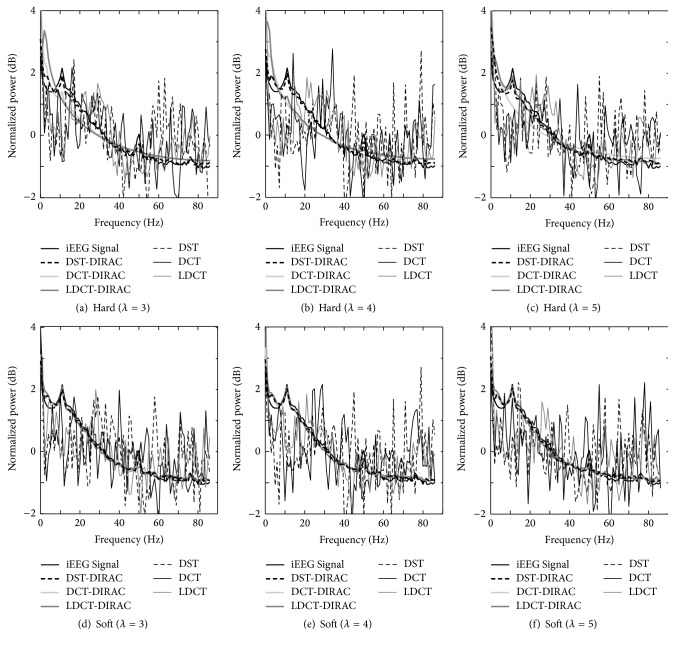
An averaged normalized FFT obtained from 100 of iEEG and combination of two morphological components and single morphological component at *λ* varies from 3 to 5 with hard and soft thresholds.

**Figure 8:**
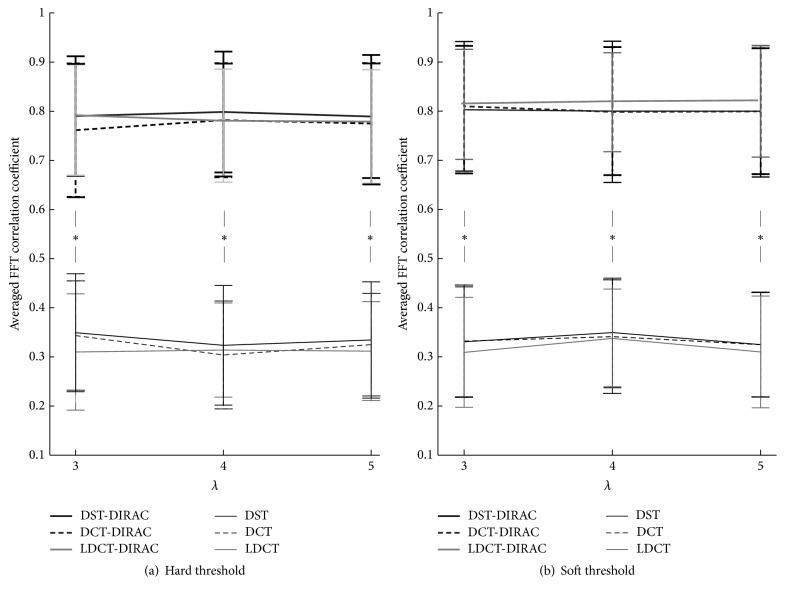
A comparison of FFT correlation coefficients between iEEG data and morphological component decomposed by explicit dictionaries. ^*∗*^Significant differences (*p* < 0.05). (a) Combined two morphological components and (b) single morphological component. Mean value and standard deviation calculated from all 100 decomposed datasets by explicit dictionaries with hard and soft thresholds.

There were similar variances and average values in the evaluation of EEG signal decomposition using the time domain, and then we introduced a measure in the frequency domain. As mentioned in Section 2.2, EEG signals carry information to represent the current brain stage in the specific tendency in the frequency domain, such as having a synchronized neural activities by showing the existence of a peak in the frequency spectrum. The EEG data used in the evaluation showed a peak around 10 Hz under the closing eye condition and a peak around 50 Hz under the opening eye condition. Therefore, in the frequency analysis, a 10 Hz peak will be an index to tell how much the reconstructed signal preserves the original information contained in the original iEEG data under closing eye condition.  Figure 7 showed the averaged normalized FFT as the comparison between three combinations of the dictionaries. Interestingly, although DST, DCT, and LDCT single components seemed to reconstruct the EEGs because of a high cc value in the time domain, the frequency spectrum analysis clarified the fact that the single component cannot reproduce the necessary tendency of EEG signals peak. Meanwhile, the combination of 2nd and 3rd components, which means oscillatory and spike components, successfully reproduced the EEG signal tendency, suggesting the importance of the spike information that presumably synchronizes background oscillatory behaviors. The 10 Hz peak can be reproduced according to parameter conditions easily. However, 50 Hz peak is difficult to be reproduced, especially for LDCT-DIRAC component in every case. In the viewpoint of the tolerance in the change of the threshold value, the soft threshold method showed the robust performance of the signal information preservation, which is consistent with the result of EOGs shown in Figure 6. As shown in Figure 8, the reconstruction accuracy of the frequency profile by two dictionaries was proved by a significant difference between the results of two morphological and single morphological components (*t*-test; *p* < 0.01 in both hard and soft thresholds). This evidence suggests the importance of the DIRAC component for EEG signals, which was not equivalent to the noise, or rather carrying some information.

### 4.3. Decomposition of EOG from Real EEG Data

#### 4.3.1. EEG Data

The real scalp EEG and EOG data were obtained from the data demonstrated in the paper written by Ai et al. (2016) [50]. These data were recorded from 23 EEG channels (FP1, FP2, F7, F3, Fz, F4, F8, FC5, FC1, FC2, FC4, T7, C3, Cz, C4, T8, CP1, CP2, P3, Pz, P4, O1, and O2) and 7 EOG channels (V1u, V1d, V2u, and V2d vertical EOG 〈VEOG〉 electrodes were placed on supraorbital and infraorbital rims of each eye; HL and HR horizontal EOG 〈HEOG〉 electrodes were on the left and right outer canthi; Vz was on the forehead approximately 25 mm above the nasion), respectively, according to 10–20 International System (BrainAmp amplifier, Brain Products GmbH) from the 8 participants seated in a comfortable armchair, with the base adjusted according to a participant height. The eyes of the participants were fixed straightly to the fixation cross in the center of the monitor screen. The stimulus was displayed by a CRT monitor. A chin support frame was used to keep the participant's head position fixed and fix their head to the supporting frame without laying their chins on the supporting bar to avoid the jaw clenching artifact. The distance between eyes and monitor was set to 70 cm. The sampling rate was 500 Hz. The whole details of the experiment protocols were given in Ai et al. (2016) [50].

#### 4.3.2. Results with Real EEG-EOG

**Figure 9:**
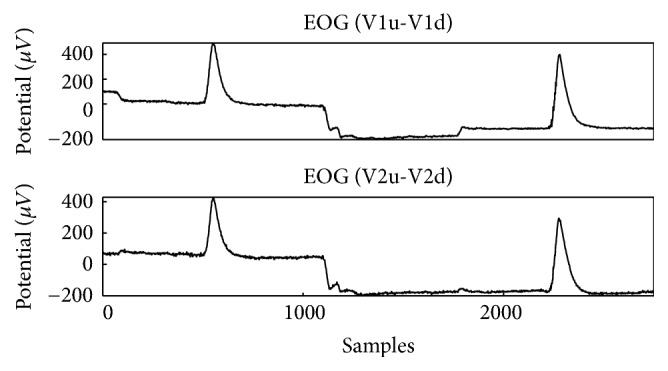
An example of EOG signal taken from right and left side of eyes.

**Figure 10:**
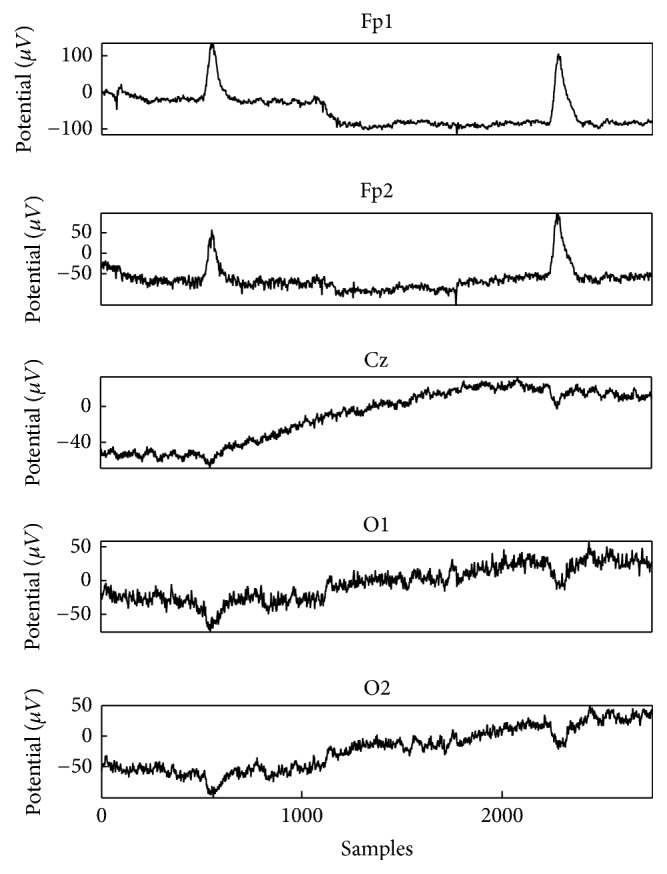
An example of real EEG signal taken from Fp1, Fp2, Cz, O1, and O2 electrode channels.

According to Ai et al. [50], the real EEG-EOG data were divided into 4 sessions. Each session had 12 tasks of eye movement. The two EOG signals were collected from “V1d-V1u” and “V2d-V2u” at right and left sides of the eye, as shown in Figure 9, and both signals showed the same kind of tendency because vertical EOG propagated symmetrically in an anterior-posterior direction.  Figure 10 showed the real EEG signals were taken from some electrodes, for example, Fp1, Fp2, Cz, O1, and O2, which represent EOG influence depending on the frontal, central, and occipital parts of the brain.

**Figure 11:**
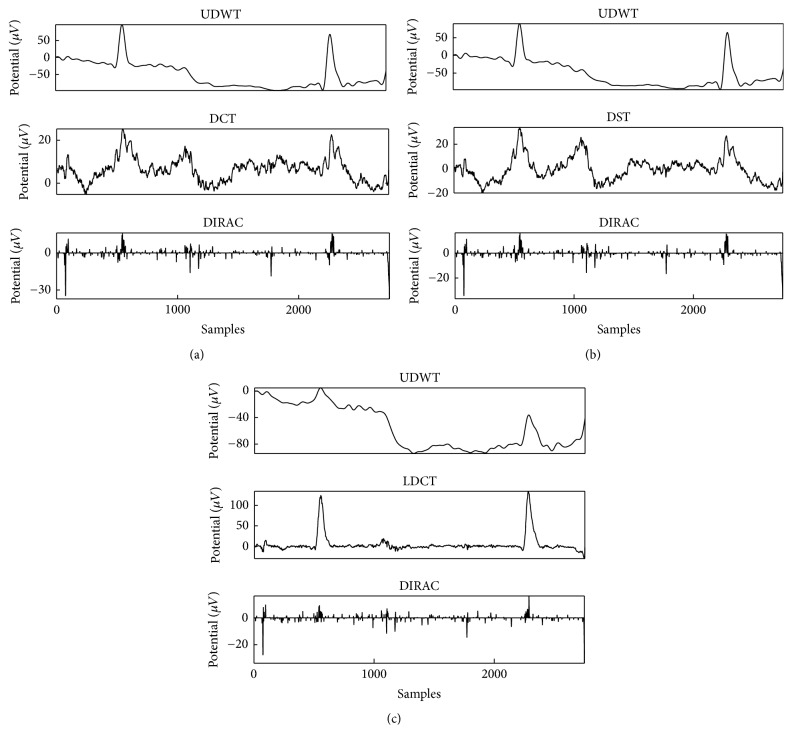
Component separated from EEG (Fp1 electrode) signal by explicit dictionaries: (a) UDWT-DCT-DIRAC, (b) UDWT-DST-DIRAC, and (c) UDWT-LDCT-DIRAC, respectively.

The selected explicit dictionaries were used to represent the targeted component for the EEG and EOG signal. EEG and EOG were distinguished based on the morphology that was observed in the EEG and EOG. The lateral eye movements mostly affect frontal electrodes [45]. Therefore, Fp1 electrode was used to decompose and demonstrate the effectiveness of our proposed method with MCA as showed in Figure 11 and the same method was applied to all the 23 electrodes. All EEG signals were morphologically decomposed with redundant transform.

**Figure 12:**
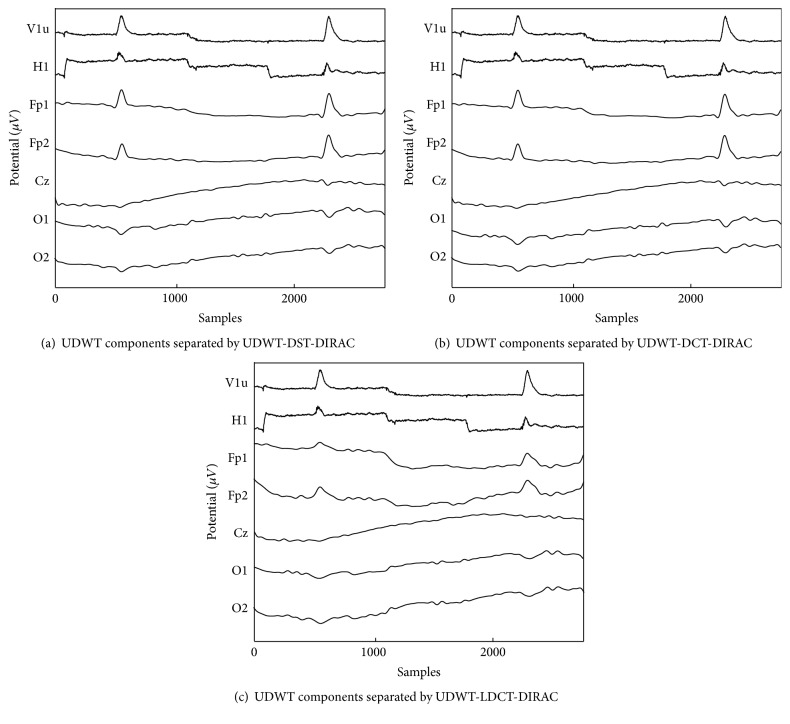
UDWT component taken from Fp1, Fp2, Cz, O1, and O2 separated by UDWT-DCT-DIRAC, UDWT-DST-DIRAC, and UDWT-LDCT-DIRAC, respectively.

Figure 11(a) demonstrated the decomposition of components by the first explicit dictionary; it was divided into three different morphologies of the EEG signal. Figures 11(b) and 11(c) showed the second and the third explicit dictionary of redundant transform, respectively. The overcomplete dictionary was a combination of redundant transforms that characterized the component in a different morphology. Accordingly, one of redundant transforms can be differentiated into decomposed components by overcomplete dictionaries. The first component was decomposed by “UDWT” of each overcomplete dictionary and was analyzed via the slow and blink type morphology. The second component was decomposed by “DCT”; “DST”; and “LDCT” and was analyzed via the background of the signal which was similar to the EEG signal and the third component was decomposed by “DIRAC” and was analyzed via the unexpected spike. The first overcomplete dictionary decomposed the EEG signal without changing the monomorphic, polymorphic, and transient properties. The cc between the original signal and the summation of all decomposed components was close to one.  Figure 12 showed the raw EOG signal taken from the vertical and horizontal channel and first decomposed component taken from Fp1, Fp2, Cz, O1, and O2, respectively.

Table 1 showed individual cc of original EEG signals and recomposed EEG signals from the combination of components with respect to different channels and combinations of dictionaries.  Table 2 showed the cc between filter raw EOG signal taken from vertical and horizontal channels and decomposed first component from Fp1, Fp2, Cz, O1, and O2, respectively.

## 5. Discussion

Artifact contamination in EEG signal has been a common important issue in neurobiological event diagnosis and neuroscientific research. The various methods were applied to remove the artifacts from EEG [2, 11–13, 21, 23, 24, 26–28, 52]. The decomposition based analysis was used in removal of EOG artifacts in EEG [22, 27, 28, 30, 40, 42]. However, those methods lack the elucidation of what the nature of EEG signals is in the viewpoint of the signal analysis, and a systematic approach was required by treating the sparsity and nonlinearity of the signal in the time domain.

This study revealed the nature of EEG signals in the sense of morphologies contained in the original signal, by using MCA. The UDWT was used to decompose the slow and bump morphology. The DCT, DST, and LDCT transform were used to decompose the EEG signal. Spike type morphology was decomposed by DIRAC. Redundant transforms of DCT and DST have a similar capability in representing the morphology of oscillatory activities. Therefore, we used the DCT, LDCT, and DST dictionaries for validations of EEG signal. The detailed significant differences in morphology of DCT and DST were given in past studies [56, 57], while in our analysis there were no significant differences. The right combination of redundant transforms to form overcomplete dictionary revealed the desired decomposition in principle.

In Section 4.2, “Blocks,” “Bumps” similar to simulated EOG signal defined in past studies [59–61] as shown in Figure 4 and EEG data [58], were used to validate our purposed method. The “Blocks” with abrupt changes were similar to the horizontal and vertical eye movements simply as described by past studies [25, 51]. For the sake of simplicity in the present study, “Bumps” were used as a representative signal form as eye blinks that happens in unexpected timings as illustrated in Figure 5. The separation of components by given dictionary works well in this evaluation but the further analysis is necessary for the evaluation of the signal decomposition with complex eye movements, which requires presumably various redundant dictionaries. In the verification of the component discrimination as shown in Figures 6 and 7, the accuracy of the averaged EOG component decomposition was above 90%, which suggests a plausible performance even in the complex eye movements. The combined DST and DIRAC dictionaries had better decomposition performance than others, while DST and DCT theoretically have no meaningful difference. The usage of iEEG as the true EEG signal had a large benefit, which can be used for the performance test for past proposed methods, like ICA and PCA consistently. Our proposed method successfully demonstrated the performance in cc and the frequency profile especially in Section 4.2, while, in the serious discussion of the real EEG and EOG signals, the DST or DCT component exhibited a baseline fluctuation of the signal which denotes the persistence of the EOG component or other slow frequency artifacts noises, and the factor will be improved by the fine-tuned design of the DST or DCT dictionary with a band pass filter function. In addition, the threshold problem exits in the optimization algorithm and number of iterations [37].

The EEG decomposition had not mark with a combination of a second and a third component of EEG signal decomposed by “DST, DCT, and LDCT” and DIRAC, respectively, based on the morphology. Therefore, the accurate combination in the further perspective will be considerable. Even the combination of all components and the mixed signal or real EEG signal had cc above 97% for all redundant transforms, as is analyzed in the frequency spectrum; the signal morphology has further meaning in the viewpoint of the signal transmission. The MCA method has such an extended and flexible availability for signal analyses.

## 6. Conclusion

Morphological Component Analysis methodology was applied to the simulated, semisimulated, and real EOG and EEG signal. The successful decomposition of the EOG and EEG signal into their morphological component was demonstrated. It seems to be that the EEG signals and artifacts in EEG have been represented by different explicit dictionaries. We analyzed the EEG signals involved with the EOG artifacts, which were influenced by task conditions. The DIRAC explicit dictionary decomposed the EEG signal into spike-like activities, which may be related to the transient property of EEG. UDWT explicit dictionary represents slow movement or bumps. DCT, DST, and LDCT explicit dictionaries represent dominant signal that represents EEGs or pure tones signals as monomorphic and polymorphic activities. These results suggested that the effectiveness of MCA in ocular artifacts removal from the EEG raw signal was derived from signal morphology characterized as slow and smooth change of EOG time series. In the further analysis, the MCA is required to compare with other competing methods for the EEG and EOG signal decomposition.

## Figures and Tables

**Table 1:** cc of original signal and sum of the decomposed components.

EEG channel	Correlation coefficient		
	UDWT-DST-DIRAC	UDWT-DCT-DIRAC	UDWT-LDCT-DIRAC
Fp1	0.9921 ± 0.014	0.9921 ± 0.014	0.9932 ± 0.013
Fp2	0.992 ± .017	0.9919 ± .018	0.9932 ± .014
Cz	0.9898 ± .01	0.9899 ± .01	0.9908 ± .009
O1	0.9836 ± .015	0.9836 ± .016	0.9869 ± .012
O2	0.9855 ± .013	0.9856 ± .013	0.9849 ± .015

**Table 2:** cc between filtered EOG and UDWT component decomposed by UDWT dictionary.

EOG channels	EEG channels				
	Fp1	Fp2	Cz	O1	O2
Correlation coefficient					
UDWT-DST-DIRAC					
V1d	0.6777	0.657	0.7267	0.9223	0.6445
V1u	0.6814	0.646	0.6606	0.9419	0.6354
V2d	0.916	0.906	0.9402	0.882	0.5402
V2u	0.2332	0.2268	0.2353	0.6388	0.8494
H1	0.8582	0.847	0.8444	0.6902	0.5839
H2	0.9419	0.9468	0.9559	0.7476	0.4752
UDWT-DCT-DIRAC					
V1d	0.6792	0.6595	0.68	0.9213	0.6352
V1u	0.6835	0.6396	0.6424	0.9581	0.6585
V2d	0.9193	0.902	0.9362	0.8764	0.5145
V2u	0.2331	0.2183	0.2316	0.6639	0.8919
H1	0.8593	0.8507	0.8626	0.6759	0.5376
H2	0.9442	0.9465	0.9662	0.7266	0.4346
UDWT-LDCT-DIRAC					
V1d	0.6794	0.6403	0.7219	0.8892	0.65
V1u	0.6443	0.6279	0.6459	0.8196	0.5381
V2d	0.9142	0.9072	0.9395	0.9592	0.6506
V2u	0.2351	0.2235	0.2383	0.3747	0.6804
H1	0.8637	0.8449	0.8471	0.7638	0.5734
H2	0.9586	0.9558	0.9577	0.8826	0.5598
